# Supplementary material for: Knowledge and motivations of training in peer review: An international cross-sectional survey
Source: PLoS One. 2023 Jul 12;18(7):e0287660. doi: 10.1371/journal.pone.0287660 (PMC10337866; doi:10.1371/journal.pone.0287660)
Supplement: S1 File — (PDF) [file pone.0287660.s001.pdf]

S1 File. Complete list of survey questions delivered through SurveyMonkey.

Demographics

#	Question	Answer
1	What is your age group?	a) 18-24<br>b) 25-34<br>c) 35-44<br>d) 45-54<br>e) 55-64<br>f) 65 +
2	What is your gender identity?	a) Man<br>b) Woman<br>c) Non-binary<br>d) Prefer to self-describe, below:<br>e) Prefer not to say
3	What country do you live in?	[drop down list]
4	Which describes you best?	a) Undergraduate student<br>b) Master's student<br>c) PhD student<br>d) Post-doctoral fellow<br>e) Independent researcher (e.g., assistant/associate/full professor)<br>f) Research support staff (e.g., research assistant, research coordinator)<br>g) Other, please specify:
5	How many years of experience do you have with scholarly publishing (i.e. writing and publishing manuscripts)?	a) < 1 year<br>b) 1-5 years<br>c) 6-10 years<br>d) 11-15 years<br>e) 16-20 years<br>f) 21 + years
6	How would you primarily describe the research you conduct?	a) Clinical<br>b) Pre-clinical ("basic" science)<br>c) Other, please specify:
7	How would you describe the institution of your primary occupation?	a) University/college<br>b) Research institute<br>c) Healthcare institution (e.g., medical centre, hospital)<br>d) Private sector (e.g., pharmaceutical company)<br>e) Not-for-profit<br>f) Government organization<br>g) Other, please specify:

8	How many articles have you peer reviewed in the last 12 months?	a) 0<br>b) 1-3<br>c) 4-6<br>d) 6-10<br>e) >10<br>f) I have never been a peer reviewer
9	For how many years have you been active as a manuscript peer reviewer?	a) < 1 year<br>b) 1-5 years<br>c) 6-10 years<br>d) 11-15 years<br>e) 16-20 years<br>f) 21 + years
10	How many peer reviewed articles have you published to date?	a) < 2<br>b) 3-5<br>c) 6-10<br>d)11-20<br>e) 21-50<br>f) 51+

Questions relating to experience with peer review

#	Question	Answer
11	Have you completed any formal training in manuscript peer review?	a) Yes → go to question 12<br>b) No → go to question 15<br>c) Unsure → go to question 15
12	What format of training best describes the training you received? Check all that apply.	a) Online lecture<br>b) Online course (at least 6 sessions)<br>c) In-person lecture<br>d) In-person half day workshop<br>e) In-person full day workshop<br>f) Shadowing a mentor/ghost-writing<br>g) Self-selected reading material<br>h) Online resources/modules<br>i) Other, please specify
13	Who provided the training you received? Check all that apply.	a) A journal<br>b) A publisher (of multiple journals)<br>c) A university/college<br>d) Private company<br>e) Unsure/don't know<br>f) Other, please specify:

14	When did you receive the training?	a) ≤1 year ago<br>b) 2 years ago<br>c) 3 years ago<br>d) 4 years ago<br>e) ≥ 5 years ago
15	Does the primary institution you are affiliated with offer formal training for peer review?	a) Yes and I have completed it → go to question 16<br>b) Yes but I have not completed it → go to question 16<br>c) No → go to next section<br>d) Unsure/don't know → go to next section
16	What type of training does your primary institution offer? Check all that apply.	a) Online lecture<br>b) Online course (at least 6 sessions)<br>c) In-person lecture<br>d) In-person half day workshop<br>e) In-person full day workshop<br>f) Shadowing a mentor/ghost-writing<br>g) Self-selected reading material<br>h) Online resources/modules<br>i) Unsure/don't know<br>j) Other, please specify

Opinion-based questions about manuscript peer review

#	Question	Answer
17	Please rate how comfortable you are with the peer review process:<br><br>The first time you did a peer review, how well prepared did you feel you were?<br><br>How well prepared do you feel you are to act as a peer reviewer currently?	*Scale of 1-7 (Very unprepared – Very prepared), N/A*
18	What skills do you think you could improve on in terms of giving a peer review? Check all that apply.	a) Time management<br>b) Structuring a review<br>c) Critical appraisal of theory<br>d) Critical appraisal of methods<br>e) Critical appraisal of statistics<br>f) Understanding of peer reviewer expectations<br>g) If asked by the journal, making a 'decision' on whether to accept/revise/reject a paper<br>h) None of the above<br>i) Other, please specify

19	Please indicate how much you agree with the following statements based on your overall experiences with peer review in the last 12 months:<br>1) Peer review is important for ensuring the quality and integrity of scholarly communication<br>2) My experience acting as a peer reviewer has been positive<br>3) My experience receiving peer review has been positive<br>4) In general, there is a lack of knowledge and understanding for how to properly conduct peer review<br>5) Peer reviewers should receive formal training in peer review prior to completing peer review assignments for journals.<br>6) My institution values that I contribute to my research field by acting as a peer reviewer<br>7) There are appropriate incentives in place to motivate me to engage in peer review	*Scale of 1-7 (Strongly disagree – Strongly agree)*, N/A<br><br>Any comments to illustrate your answer.
20	If training were to be offered, what topics would you be most interested in learning how to evaluate? Rank with most interested at the top.	a) Research question<br>b) Abstract<br>c) Study design / methodology<br>d) Statistics<br>e) References<br>f) Discussion<br>g) Study limitations<br>h) Supplementary reporting<br>i) Concerns of publication ethics (ex. Plagiarism, conflicts of interest, misconduct)<br>j) How to construct a peer review
21	What organization is best positioned to provide peer review training? Rank with most preferred at the top.	a) University / college<br>b) Membership societies<br>c) Scholarly publishers or journals<br>d) Independent course providers<br>e) It doesn't matter<br>f) Peer review training is unnecessary
22	Who should fund the peer review training? Rank in order of most preferred.	a) You (the person being trained)<br>b) Your primary research institution<br>c) Scholarly publishers<br>d) Funders<br>e) Nobody<br>f) It does not matter

23	What would be the best way to deliver peer review training? Rank in order with most preferred at the top.	a) Online lecture<br>b) Online course (at least 6 sessions)<br>c) In-person lecture<br>d) In-person half day workshop<br>e) In-person full day workshop<br>f) Shadowing a mentor/ghost-writing<br>g) Self-selected reading material<br>h) Online resources/modules

Journal perspective

#	Question	Answer
24	Do you operate or work for a journal that publishes peer reviewed articles?	a) Yes, continue to this section<br>b) No, skip this section
25	What is your role at the journal?	a) Editor in chief<br>b) Editorial board member<br>c) Other, please specify
26	Does the journal have explicit eligibility criteria for selecting peer reviewers?	a) Yes<br>b) No<br>c) Unsure/Don't know
27	Does the journal require any explicit training prior to allowing peer reviewers to assess a manuscript?	a) Yes, internally provided training → skip to 29<br>b) Yes, externally provided training → to 28<br>c) No → skip to 30<br>d) Unsure/don't know → skip to 30<br>e) Other (please specify) → skip to 28
28	Who provides the training? Provide a link if possible.	[comment box]
29	What type of training is required? Check all that apply.	a) Online lecture<br>b) Online course (at least 6 sessions)<br>c) In-person lecture<br>d) In-person half day workshop<br>e) In-person full day workshop<br>f) Shadowing a mentor/ghost-writing<br>g) Self-selected reading material<br>h) Online resources/modules<br>i) Other, please specify
30	How many hours of peer review training does the journal require before allowing reviewers to assess a manuscript?	a) No set amount of training<br>b) 1-5 hours<br>c) 6-10 hours<br>d) 11-15 hours<br>e) 15-20 hours<br>f) 20 hours +<br>g) Other (please specify)

31	Does the journal explicitly assess peer review reports of new peer reviewers?	a) No<br>b) Unsure/Don't know<br>c) Yes (please specify how):
32	Does the journal have a database of peer reviewers?	a) Yes, less than 50 reviewers<br>b) Yes, more than 50 reviewers<br>c) No<br>d) Unsure/Don't know
33	Does the journal explicitly provide reporting guidelines to reviewers as part of the peer review assessment process?	a) Yes<br>b) No<br>c) Unsure/Don't know

Open-ended

1. Other than training, how do you believe the quality of peer review could be improved?

2. What barriers do you face in engaging in peer review training?

3. What would incentivize you to obtain additional training in peer review best practices?

4. Any other comments you wish to share relating to peer review training:
