# Supplementary material for: Knowledge and motivations of training in peer review: An international cross-sectional survey
Source: PLoS One. 2023 Jul 12;18(7):e0287660. doi: 10.1371/journal.pone.0287660 (PMC10337866; doi:10.1371/journal.pone.0287660)
Supplement: S1 Table — (PDF) [file pone.0287660.s003.pdf]

Corresponding author emails extracted from recent articles published in biomedical journals; emailed survey invitation
(n = 2,000)

Emails sent/received as of June 6, 2022
(n = 1,861)

Survey responses as of June 13, 2022
(n = 186)

Emails unable to be sent, with reason
(n = 107):

"550 delivery not authorized, message refused" (n = 6)
"550 5.4.1 Recipient address rejected: Access denied" (n = 7)
"Connection is not secured by TLS" (n = 1)
"Unable to look up host [x]: Name or service not known" (n = 7)
"550 Ip frequency limited" (n = 11)
"The email that you tried to reach is disabled" (n = 1)
"Mailbox full – quota exceeded" (n = 4)
"550 5.1.0 Address rejected" (n = 7)
"Requested mail action aborted, mailbox not found" (n = 2)
"Service unavailable; Client host [137.122.8.106] blocked using blackholes.easynet.nl" (n = 1)
"553 5.7.1 Sender ERROR" (n = 1)
"Requested actions not taken as the mailbox is unavailable" (n = 2)
"554 Security violation" (n = 1)
"Temporary unavailable user" (n = 1)
"Message cannot be accepted, spam rejection" (n = 1)
"The email address you entered couldn't be found" (n = 6)
"Recipient address rejected: User unknown" (n = 4)
"554 5.7.1. Recipient address rejected: Invalid-Recipient" (n = 1)
"The email account that you tried to reach does not exist" (n = 3)
"550 5.1.1 Error: invalid recipients is found" (n = 1)
"511 no mailbox here by that name" (n = 1)
"550 5.0.0 Unrouteable address" (n = 1)
"451 4.1.0 Recipient disabled" (n = 1)
"Connection refused" (n = 2)
Email address provided doesn't allow for email to be sent (n = 3)
"Connection timed out" (n = 3)
Mail rejected from recipient (n = 3)
"Part of [recipient's] network is on our block list (S3140)" (n = 22)
"Recipient's mailbox is undergoing maintenance" (n = 1)
"The email admin for the organization [x] created an email rule restriction" (n = 1)
"550 5.7.107 Recipient not on bypass list, your IP has been found on a block list" (n = 1)
"550 Envelope blocked" (n = 1)

Unable to reach/participate, with reason
(n = 32):

Unable to participate in study (replied) (n = 19)
Out of office for duration of study (n = 9)
No longer working in their department/institution, no new email provided (n = 4)
