# Supplementary material for: Knowledge and motivations of training in peer review: An international cross-sectional survey
Source: PLoS One. 2023 Jul 12;18(7):e0287660. doi: 10.1371/journal.pone.0287660 (PMC10337866; doi:10.1371/journal.pone.0287660)
Supplement: S2 Table — (PDF) [file pone.0287660.s004.pdf]

What is your age group?

		Frequency	Percent	Valid Percent	Cumulative Percent
Valid	18-24	1	.6	.6	.6
	25-34	30	17.4	17.5	18.1
	35-44	60	34.9	35.1	53.2
	45-54	35	20.3	20.5	73.7
	55-64	26	15.1	15.2	88.9
	65+	19	11.0	11.1	100.0
	Total	171	99.4	100.0	
Missing	System	1	.6		
Total		172	100.0		

What is your gender identity?

		Frequency	Percent	Valid Percent	Cumulative Percent
Valid	Man	97	56.4	57.1	57.1
	Woman	73	42.4	42.9	100.0
	Total	170	98.8	100.0	
Missing	System	2	1.2		
Total		172	100.0		

What country do you live in?

		Frequency	Percent	Valid Percent	Cumulative Percent
Valid	United States	41	23.8	24.0	24.0
	United Kingdom	13	7.6	7.6	31.6
	Canada	11	6.4	6.4	38.0
	Australia	4	2.3	2.3	40.4
	Argentina	2	1.2	1.2	41.5

Bangladesh	1	.6	.6	42.1
Brazil	4	2.3	2.3	44.4
Switzerland	3	1.7	1.8	46.2
Germany	1	.6	.6	46.8
Denmark	1	.6	.6	47.4
Egypt	2	1.2	1.2	48.5
Spain	1	.6	.6	49.1
Ethiopia	3	1.7	1.8	50.9
France	2	1.2	1.2	52.0
Croatia	3	1.7	1.8	53.8
Indonesia	1	.6	.6	54.4
India	13	7.6	7.6	62.0
Iraq	3	1.7	1.8	63.7
Iran	3	1.7	1.8	65.5
Italy	7	4.1	4.1	69.6
Japan	2	1.2	1.2	70.8
Kenya	1	.6	.6	71.3
Lebanon	1	.6	.6	71.9
Mexico	3	1.7	1.8	73.7
Nigeria	2	1.2	1.2	74.9
Netherlands	2	1.2	1.2	76.0
Nepal	4	2.3	2.3	78.4
New Zealand	1	.6	.6	78.9
Philippines	5	2.9	2.9	81.9
Pakistan	2	1.2	1.2	83.0
Poland	1	.6	.6	83.6
West Bank	1	.6	.6	84.2
Portugal	2	1.2	1.2	85.4
Romania	1	.6	.6	86.0
Rwanda	3	1.7	1.8	87.7
Saudi Arabia	2	1.2	1.2	88.9
Sudan	1	.6	.6	89.5
Sweden	2	1.2	1.2	90.6
Singapore	2	1.2	1.2	91.8
Slovenia	1	.6	.6	92.4
Turks and Caicos Islands	1	.6	.6	93.0
Thailand	1	.6	.6	93.6
Tunisia	3	1.7	1.8	95.3

	Turkey	1	.6	.6	95.9
	Ukraine	1	.6	.6	96.5
	Uganda	1	.6	.6	97.1
	South Africa	4	2.3	2.3	99.4
	Zimbabwe	1	.6	.6	100.0
	Total	171	99.4	100.0	
Missing	System	1	.6		
Total		172	100.0		

Which describes you best? (Occupation/Position)

		Frequency	Percent	Valid Percent	Cumulative Percent
Valid	Other (please specify)	22	12.8	12.8	12.8
	Master's student	10	5.8	5.8	18.6
	PhD student	12	7.0	7.0	25.6
	Post-doctoral fellow	14	8.1	8.1	33.7
	Independent researcher (e.g. assistant/associate/full professor)	108	62.8	62.8	96.5
	Research support staff (e.g. research assistant, research coordinator)	6	3.5	3.5	100.0
	Total	172	100.0	100.0	

How many years of experience do you have with scholarly publishing (i.e. writing and publishing manuscripts)?

		Frequency	Percent	Valid Percent	Cumulative Percent
Valid	< 1 year	1	.6	.6	.6
	1-5 years	38	22.1	22.1	22.7

		Frequency	Percent	Valid Percent	Cumulative Percent
	6-10 years	44	25.6	25.6	48.3
	11-15 years	29	16.9	16.9	65.1
	16-20 years	13	7.6	7.6	72.7
	21+ years	47	27.3	27.3	100.0
	Total	172	100.0	100.0	

How would you primarily describe the research you conduct?

		Frequency	Percent	Valid Percent	Cumulative Percent
Valid	Other (please specify)	58	33.7	34.1	34.1
	Clinical	82	47.7	48.2	82.4
	Pre-clinical ("Basic science")	30	17.4	17.6	100.0
	Total	170	98.8	100.0	
Missing	System	2	1.2		
Total		172	100.0		

How would you describe the institution of your primary occupation?

		Frequency	Percent	Valid Percent	Cumulative Percent
Valid	Other (please specify)	9	5.2	5.3	5.3
	University/college	103	59.9	60.6	65.9
	Research institute	4	2.3	2.4	68.2
	Healthcare institution (e.g. medical centre, hospital)	42	24.4	24.7	92.9
	Private sector (e.g. pharmaceutical company)	4	2.3	2.4	95.3

		Frequency	Percent	Valid Percent	Cumulative Percent
	Not-for-profit	1	.6	.6	95.9
	Government organization	7	4.1	4.1	100.0
	Total	170	98.8	100.0	
Missing	System	2	1.2		
Total		172	100.0		

How many articles have you peer reviewed in the last 12 months?

		Frequency	Percent	Valid Percent	Cumulative Percent
Valid	0	7	4.1	4.1	4.1
	1-3	41	23.8	24.0	28.1
	4-6	38	22.1	22.2	50.3
	6-10	23	13.4	13.5	63.7
	>10	58	33.7	33.9	97.7
	I have never been a peer reviewer	4	2.3	2.3	100.0
	Total	171	99.4	100.0	
Missing	System	1	.6		
Total		172	100.0		

For how many years have you been active as a manuscript peer reviewer?

		Frequency	Percent	Valid Percent	Cumulative Percent
Valid	< 1 year	11	6.4	6.5	6.5
	1-5 years	59	34.3	34.9	41.4
	6-10 years	43	25.0	25.4	66.9
	11-15 years	15	8.7	8.9	75.7
	16-20 years	13	7.6	7.7	83.4
	21 + years	28	16.3	16.6	100.0
	Total	169	98.3	100.0	

Missing	System	3	1.7		
Total		172	100.0		

How many peer reviewed articles have you published to date?

		Frequency	Percent	Valid Percent	Cumulative Percent
Valid	< 2	5	2.9	2.9	2.9
	3-5	16	9.3	9.3	12.2
	6-10	22	12.8	12.8	25.0
	11-20	23	13.4	13.4	38.4
	21-50	36	20.9	20.9	59.3
	51+	70	40.7	40.7	100.0
	Total	172	100.0	100.0	

Have you completed any formal training in peer review?

		Frequency	Percent	Valid Percent	Cumulative Percent
Valid	Yes	26	15.1	15.2	15.2
	No	144	83.7	84.2	99.4
	Unsure	1	.6	.6	100.0
	Total	171	99.4	100.0	
Missing	System	1	.6		
Total		172	100.0		

What type of formal training received?

		Responses		
		N	Percent	Percent of Cases
What type of formal training received?	Online lecture	10	16.4%	37.0%
	Online course (at least 6 sessions)	10	16.4%	37.0%
	In-person lecture	12	19.7%	44.4%
	In-person half day workshop	2	3.3%	7.4%
	In-person full day workshop	7	11.5%	25.9%
	Shawdowing a mentor/ghost-writing	4	6.6%	14.8%
	Self-selected reading material	7	11.5%	25.9%
	Online resource/modules	8	13.1%	29.6%
	Other	1	1.6%	3.7%
Total		61	100.0%	225.9%

Who provided the training you received?

		Responses		
		N	Percent	Percent of Cases
Who provided the traning you received?	A journal	4	12.1%	14.8%
	A publisher	6	18.2%	22.2%
	A university/college	18	54.5%	66.7%
	Private company	2	6.1%	7.4%
	Unsure/Don't know	2	6.1%	7.4%
	Other	1	3.0%	3.7%

Total		33	100.0%	122.2%

When did you receive the training?

		Frequency	Percent	Valid Percent	Cumulative Percent
Valid	≤1 year ago	4	2.3	14.8	14.8
	2 years ago	4	2.3	14.8	29.6
	3 years ago	6	3.5	22.2	51.9
	4 years ago	2	1.2	7.4	59.3
	≥5 years ago	11	6.4	40.7	100.0
	Total	27	15.7	100.0	
Missing	System	145	84.3		
Total		172	100.0		

Does the primary institution you are affiliated with offer formal training for peer review?

		Frequency	Percent	Valid Percent	Cumulative Percent
Valid	Yes and I have completed it	10	5.8	5.8	5.8
	Yes but I have not completed it	5	2.9	2.9	8.8
	No	108	62.8	63.2	71.9
	Unsure/don't know	48	27.9	28.1	100.0
	Total	171	99.4	100.0	
Missing	System	1	.6		
Total		172	100.0		

Type of training your institution offers

		Responses		
		N	Percent	Percent of Cases
Type of training your institution offers	Online lecture	6	20.7%	37.5%
	Online course (at least 6 sessions)	2	6.9%	12.5%
	In-person lecture	3	10.3%	18.8%
	In-person half day workshop	3	10.3%	18.8%
	In-person full day workshop	5	17.2%	31.3%
	Shadowing a mentor/ghost-writing	2	6.9%	12.5%
	Self-selected reading material	2	6.9%	12.5%
	Online resource/modules	4	13.8%	25.0%
	Unsure/Don't know	1	3.4%	6.3%
	Other	1	3.4%	6.3%
Total		29	100.0%	181.3%

The first time you did a peer review, how well prepared did you feel you were?

		Frequency	Percent	Valid Percent	Cumulative Percent
Valid	Very unprepared	18	10.5	10.8	10.8
	Unprepared	40	23.3	24.1	34.9
	Slightly unprepared	30	17.4	18.1	53.0
	Neutral/Unsure	11	6.4	6.6	59.6
	Slightly prepared	34	19.8	20.5	80.1
	Prepared	25	14.5	15.1	95.2
	Very prepared	8	4.7	4.8	100.0
	Total	166	96.5	100.0	

Missing	0	6	3.5		
Total		172	100.0		

How well prepared do you feel you are to act as a peer reviewer currently?

		Frequency	Percent	Valid Percent	Cumulative Percent
Valid	Very unprepared	2	1.2	1.2	1.2
	Unprepared	5	2.9	3.0	4.2
	Slightly unprepared	5	2.9	3.0	7.2
	Neutral/Unsure	5	2.9	3.0	10.2
	Slightly prepared	25	14.5	15.0	25.1
	Prepared	66	38.4	39.5	64.7
	Very prepared	59	34.3	35.3	100.0
	Total	167	97.1	100.0	
Missing	0	2	1.2		
	System	3	1.7		
	Total	5	2.9		
Total		172	100.0		

Skills you can improve in peer review

		Responses		Percent of Cases
		N	Percent	
Skills you can improve in peer review	None of the above	14	3.0%	8.1%
	Time Management	54	11.5%	31.4%
	Structing a review	67	14.3%	39.0%
	Critical appraisal of theory	60	12.8%	34.9%
	Critical appraisal of methods	68	14.5%	39.5%
	Critical appraisal of statistics	94	20.0%	54.7%
	Understanding of peer reviewer	59	12.6%	34.3%

	expectations			
	If asked by the journal, making a 'decision' on whether to accept/revise/reject a paper	44	9.4%	25.6%
	Other (please specify)	10	2.1%	5.8%
Total		470	100.0%	273.3%

Peer review is important for ensuring the quality and integrity of scholarly communication

		Frequency	Percent	Valid Percent	Cumulative Percent
Valid	Strongly disagree	3	1.7	1.8	1.8
	Neutral/Unsure	4	2.3	2.4	4.1
	Slightly agree	15	8.7	8.8	12.9
	Agree	54	31.4	31.8	44.7
	Strongly agree	94	54.7	55.3	100.0
	Total	170	98.8	100.0	
Missing	0	1	.6		
	System	1	.6		
	Total	2	1.2		
Total		172	100.0		

My experience acting as a peer reviewer has been positive

		Frequency	Percent	Valid Percent	Cumulative Percent
Valid	Disagree	3	1.7	1.8	1.8
	Slightly disagree	1	.6	.6	2.4
	Neutral/Unsure	13	7.6	7.9	10.4
	Slightly agree	31	18.0	18.9	29.3
	Agree	72	41.9	43.9	73.2

		Frequency	Percent	Valid Percent	Cumulative Percent
	Strongly agree	44	25.6	26.8	100.0
	Total	164	95.3	100.0	
Missing	0	3	1.7		
	System	5	2.9		
	Total	8	4.7		
Total		172	100.0		

My experience receiving peer review has been positive

		Frequency	Percent	Valid Percent	Cumulative Percent
Valid	Strongly disagree	2	1.2	1.2	1.2
	Disagree	8	4.7	4.8	6.0
	Slightly disagree	9	5.2	5.4	11.4
	Neutral/Unsure	17	9.9	10.2	21.6
	Slightly agree	32	18.6	19.2	40.7
	Agree	84	48.8	50.3	91.0
	Strongly agree	15	8.7	9.0	100.0
	Total	167	97.1	100.0	
Missing	0	1	.6		
	System	4	2.3		
	Total	5	2.9		
Total		172	100.0		

In general, there is a lack of knowledge and understanding for how to properly conduct peer review

		Frequency	Percent	Valid Percent	Cumulative Percent
Valid	Strongly disagree	2	1.2	1.2	1.2
	Disagree	14	8.1	8.3	9.5

		Frequency	Percent	Valid Percent	Cumulative Percent
	Slightly disagree	12	7.0	7.1	16.7
	Neutral/Unsure	21	12.2	12.5	29.2
	Slightly agree	43	25.0	25.6	54.8
	Agree	54	31.4	32.1	86.9
	Strongly agree	22	12.8	13.1	100.0
	Total	168	97.7	100.0	
Missing	System	4	2.3		
Total		172	100.0		

Peer reviewers should receive formal training in peer review prior to completing peer review assignments for journals

		Frequency	Percent	Valid Percent	Cumulative Percent
Valid	Strongly disagree	4	2.3	2.4	2.4
	Disagree	9	5.2	5.3	7.7
	Slightly disagree	8	4.7	4.7	12.4
	Neutral/Unsure	20	11.6	11.8	24.3
	Slightly agree	29	16.9	17.2	41.4
	Agree	58	33.7	34.3	75.7
	Strongly agree	41	23.8	24.3	100.0
	Total	169	98.3	100.0	
Missing	System	3	1.7		
Total		172	100.0		

My institution values that I contribute to my research field by acting as a peer reviewer

		Frequency	Percent	Valid Percent	Cumulative Percent
Valid	Strongly disagree	23	13.4	13.9	13.9

		Frequency	Percent	Valid Percent	Cumulative Percent
	Disagree	30	17.4	18.1	31.9
	Slightly disagree	6	3.5	3.6	35.5
	Neutral/Unsure	36	20.9	21.7	57.2
	Slightly agree	20	11.6	12.0	69.3
	Agree	31	18.0	18.7	88.0
	Strongly agree	20	11.6	12.0	100.0
	Total	166	96.5	100.0	
Missing	0	1	.6		
	System	5	2.9		
	Total	6	3.5		
Total		172	100.0		

My institution values that I contribute to my research field by acting as a peer reviewer

		Frequency	Percent	Valid Percent	Cumulative Percent
Valid	Strongly disagree	23	13.4	13.9	13.9
	Disagree	30	17.4	18.1	31.9
	Slightly disagree	6	3.5	3.6	35.5
	Neutral/Unsure	36	20.9	21.7	57.2
	Slightly agree	20	11.6	12.0	69.3
	Agree	30	17.4	18.1	87.3
	Strongly agree	21	12.2	12.7	100.0
	Total	166	96.5	100.0	
Missing	0	1	.6		
	System	5	2.9		
	Total	6	3.5		
Total		172	100.0		

There are appropriate incentives in place to motivate me to engage in peer review

		Frequency	Percent	Valid Percent	Cumulative Percent
Valid	Strongly disagree	41	23.8	24.6	24.6
	Disagree	45	26.2	26.9	51.5
	Slightly disagree	22	12.8	13.2	64.7
	Neutral/Unsure	22	12.8	13.2	77.8
	Slightly agree	18	10.5	10.8	88.6
	Agree	14	8.1	8.4	97.0
	Strongly agree	5	2.9	3.0	100.0
	Total	167	97.1	100.0	
Missing	0	2	1.2		
	System	3	1.7		
	Total	5	2.9		
Total		172	100.0		

Topics to be covered in PR training

		Responses		Percent of Cases
		N	Percent	
Topics to be covered in PR training	Research question	28	17.5%	17.5%
	Abstract	1	0.6%	0.6%
	Study design / methodology	35	21.9%	21.9%
	Statistics	33	20.6%	20.6%
	References	2	1.3%	1.3%
	Discussion	3	1.9%	1.9%
	Study limitations	2	1.3%	1.3%
	Supplementary reporting	4	2.5%	2.5%
	Concerns of publication ethics (ex. plagiarism, conflicts of interest, misconduct)	11	6.9%	6.9%
	How to construct a peer review	41	25.6%	25.6%
Total		160	100.0%	100.0%

Who is best to offer PR training?

		Responses		
		N	Percent	Percent of Cases
Who is best to offer PR training?	University / college	59	36.6%	36.6%
	Membership societies	13	8.1%	8.1%
	Scholarly publishers or journals	65	40.4%	40.4%
	Independent course providers	6	3.7%	3.7%
	It doesn't matter	14	8.7%	8.7%
	Peer review training is unnecessary	4	2.5%	2.5%
Total		161	100.0%	100.0%

Who should fund PR training?

Commented [HK1]: Also broke these down into individual frequencies - see below.

		Responses		
		N	Percent	Percent of Cases
Who should fund PR training?	You (the person being trained)	12	7.3%	7.3%
	Your primary research institution	48	29.3%	29.3%
	Scholarly publishers	79	48.2%	48.2%
	Funders	10	6.1%	6.1%
	Nobody	12	7.3%	7.3%
	It does not matter	3	1.8%	1.8%
Total		164	100.0%	100.0%

You (the person being trained)

		Frequency	Percent	Valid Percent	Cumulative Percent
Valid	1	12	7.0	9.3	9.3
	2	13	7.6	10.1	19.4
	3	19	11.0	14.7	34.1
	4	34	19.8	26.4	60.5
	5	19	11.0	14.7	75.2
	6	32	18.6	24.8	100.0
	Total	129	75.0	100.0	
Missing	System	43	25.0		
Total		172	100.0		

Your primary research institution

		Frequency	Percent	Valid Percent	Cumulative Percent
Valid	1	48	27.9	32.7	32.7
	2	45	26.2	30.6	63.3
	3	38	22.1	25.9	89.1
	4	8	4.7	5.4	94.6
	5	4	2.3	2.7	97.3
	6	4	2.3	2.7	100.0
	Total	147	85.5	100.0	
Missing	System	25	14.5		
Total		172	100.0		

Scholarly publishers

		Frequency	Percent	Valid Percent	Cumulative Percent
Valid	1	79	45.9	49.1	49.1
	2	46	26.7	28.6	77.6
	3	22	12.8	13.7	91.3
	4	10	5.8	6.2	97.5
	5	1	.6	.6	98.1
	6	3	1.7	1.9	100.0
	Total	161	93.6	100.0	

Missing	System	11	6.4		
Total		172	100.0		

Funders

		Frequency	Percent	Valid Percent	Cumulative Percent
Valid	1	10	5.8	7.1	7.1
	2	36	20.9	25.5	32.6
	3	51	29.7	36.2	68.8
	4	36	20.9	25.5	94.3
	5	5	2.9	3.5	97.9
	6	3	1.7	2.1	100.0
	Total	141	82.0	100.0	
Missing	System	31	18.0		
Total		172	100.0		

Nobody

		Frequency	Percent	Valid Percent	Cumulative Percent
Valid	1	12	7.0	9.8	9.8
	2	2	1.2	1.6	11.4
	3	3	1.7	2.4	13.8
	4	21	12.2	17.1	30.9
	5	58	33.7	47.2	78.0
	6	27	15.7	22.0	100.0
	Total	123	71.5	100.0	
Missing	System	49	28.5		
Total		172	100.0		

It does not matter

		Frequency	Percent	Valid Percent	Cumulative Percent
Valid	1	3	1.7	2.4	2.4
	2	6	3.5	4.8	7.3
	3	4	2.3	3.2	10.5
	4	19	11.0	15.3	25.8
	5	35	20.3	28.2	54.0
	6	57	33.1	46.0	100.0

	Total	124	72.1	100.0	
Missing	System	48	27.9		
Total		172	100.0		

What is best method for PR training delivery?

		Responses		
		N	Percent	Percent of Cases
What is best method for PR trng delivery	Online lecture	53	33.5%	33.5%
	Online course (at least 6 sessions)	37	23.4%	23.4%
	In-person lecture	7	4.4%	4.4%
	In-person half day workshop	8	5.1%	5.1%
	In-person full day workshop	15	9.5%	9.5%
	Shadowing a mentor/ghost-writing	17	10.8%	10.8%
	Self-selected reading material	3	1.9%	1.9%
	Online resources/modules	18	11.4%	11.4%
Total		158	100.0%	100.0%

Do you operate or work or volunteer for a journal that publishes peer reviewed articles?

		Frequency	Percent	Valid Percent	Cumulative Percent
Valid	Yes, continue to this section	79	45.9	46.7	46.7
	No, skip this section	90	52.3	53.3	100.0
	Total	169	98.3	100.0	
Missing	System	3	1.7		
Total		172	100.0		

What is your role at the journal?

		Frequency	Percent	Valid Percent	Cumulative Percent
Valid	Other (please specify)	28	16.3	33.7	33.7
	Editor in chief	7	4.1	8.4	42.2
	Editorial board member	48	27.9	57.8	100.0
	Total	83	48.3	100.0	
Missing	System	89	51.7		
Total		172	100.0		

Does the journal have explicit eligibility criteria for selecting peer reviewers?

		Frequency	Percent	Valid Percent	Cumulative Percent
Valid	Yes	31	18.0	38.3	38.3
	No	27	15.7	33.3	71.6
	Unsure/Don't know	23	13.4	28.4	100.0
	Total	81	47.1	100.0	
Missing	System	91	52.9		
Total		172	100.0		

Does the journal require any explicit training prior to allowing peer reviewers to assess a manuscript?

		Frequency	Percent	Valid Percent	Cumulative Percent
Valid	Yes, internally provided training	8	4.7	10.0	10.0
	Yes, externally provided training	2	1.2	2.5	12.5
	No	55	32.0	68.8	81.3
	Unsure/Don't know	15	8.7	18.8	100.0

	Total	80	46.5	100.0	
Missing	System	92	53.5		
Total		172	100.0		

What type of PR training be required?

		Responses		
		N	Percent	Percent of Cases
What type of PR trng be required?	Online lecture	6	22.2%	54.5%
	Online course (at least 6 sessions)	6	22.2%	54.5%
	In-person lecture	3	11.1%	27.3%
	In-person halfday workshop	1	3.7%	9.1%
	In-person fullday workshop	3	11.1%	27.3%
	Shadowing a mentor/ghost-writing	2	7.4%	18.2%
	Self-selected reading material	1	3.7%	9.1%
	Online resource/modules	4	14.8%	36.4%
	Other (please specify)	1	3.7%	9.1%
Total		27	100.0%	245.5%

How many hours of peer review training does the journal require before allowing reviewers assess a manuscript?

		Frequency	Percent	Valid Percent	Cumulative Percent
Valid	Other (please specify)	1	.6	9.1	9.1
	No set amount of training	3	1.7	27.3	36.4
	1-5 hours	2	1.2	18.2	54.5
	6-10 hours	3	1.7	27.3	81.8

	15-20 hours	1	.6	9.1	90.9
	20 hours +	1	.6	9.1	100.0
	Total	11	6.4	100.0	
Missing	System	161	93.6		
Total		172	100.0		

Does the journal explicitly assess peer review reports of new peer reviewers?

		Frequency	Percent	Valid Percent	Cumulative Percent
Valid	Yes (please specify how)	8	4.7	10.0	10.0
	No	21	12.2	26.3	36.3
	Unsure/Don't know	51	29.7	63.7	100.0
	Total	80	46.5	100.0	
Missing	System	92	53.5		
Total		172	100.0		

Does the journal have a database of peer reviewers?

		Frequency	Percent	Valid Percent	Cumulative Percent
Valid	Yes, less than 50 reviewers	13	7.6	16.3	16.3
	Yes, more than 50 reviewers	44	25.6	55.0	71.3
	No	3	1.7	3.8	75.0
	Unsure/don't know	20	11.6	25.0	100.0
	Total	80	46.5	100.0	
Missing	System	92	53.5		
Total		172	100.0		

Does the journal explicitly provide reporting guidelines to reviewers as part of the peer review assessment process?

		Frequency	Percent	Valid Percent	Cumulative Percent

Valid	Yes	51	29.7	64.6	64.6
	No	21	12.2	26.6	91.1
	Unsure/Don't know	7	4.1	8.9	100.0
	Total	79	45.9	100.0	
Missing	System	93	54.1		
Total		172	100.0		
