# Supplementary material for: Knowledge and motivations of training in peer review: An international cross-sectional survey
Source: PLoS One. 2023 Jul 12;18(7):e0287660. doi: 10.1371/journal.pone.0287660 (PMC10337866; doi:10.1371/journal.pone.0287660)
Supplement: Attachment — Submitted filename: Response to Reviewers 2.docx [file pone.0287660.s006.docx]

Figure 2 is now easier to read; however, the description incorrectly identifies the most desired training formats as all online. According to the new figure 2, the online resources/modules were ranked 5th after in person half day workshop and in person lecture.

"The most desired training formats were all online, including online lectures, online courses (at least 6 sessions),

online lectures, and online resources or modules."

The discussion now relates much more to the study findings but ends quite abruptly. I wonder whether a concluding statement would be beneficial; however, I do appreciate that this may be personal preference.

Appendix 2 needs to be renamed Appendix 1; similar for supplemental 4 to 3.

We thank the reviewer for their follow-up comments. We have edited the statement and removed online resources and modules. We have added a concluding paragraph.

Appendices and supplemental materials have been renamed.
